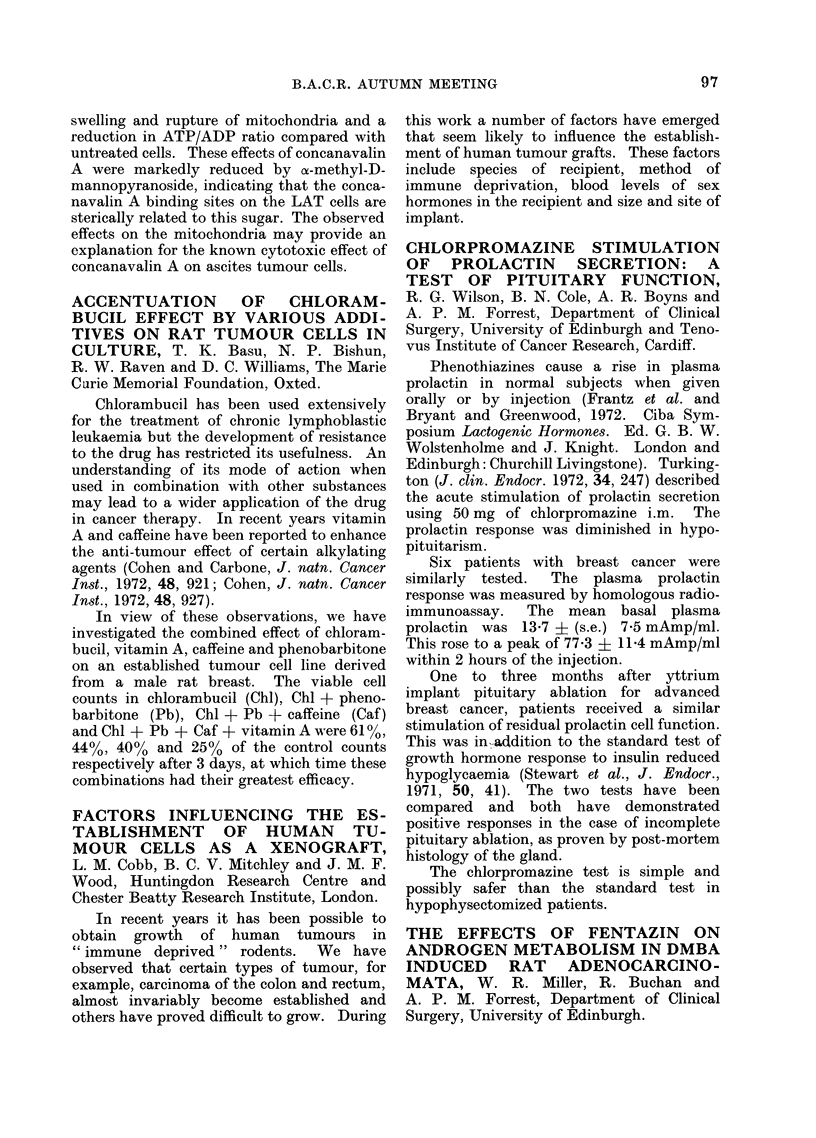# Proceedings: The effects of fentazin on androgen metabolism in DMBA induced rat adenocarcinomata.

**DOI:** 10.1038/bjc.1974.32

**Published:** 1974-01

**Authors:** W. R. Miller, R. Buchan, A. P. Forrest


**CHLORPROMAZINE STIMULATION OF PROLACTIN SECRETION: A TEST OF PITUITARY FUNCTION,** R. G. Wilson, B. N. Cole, A. R. Boyns and A. P. M. Forrest, Department of Clinical Surgery, University of Edinburgh and Tenovus Institute of Cancer Research, Cardiff.

Phenothiazines cause a rise in plasma prolactin in normal subjects when given orally or by injection (Frantz *et al.* and Bryant and Greenwood, 1972. Ciba Symposium *Lactogenic Hormones*. Ed. G. B. W. Wolstenholme and J. Knight. London and Edinburgh: Churchill Livingstone). Turkington (*J. clin. Endocr.* 1972, **34**, 247) described the acute stimulation of prolactin secretion using 50 mg of chlorpromazine i.m. The prolactin response was diminished in hypopituitarism.

Six patients with breast cancer were similarly tested. The plasma prolactin response was measured by homologous radioimmunoassay. The mean basal plasma prolactin was $13{\cdot}7 \pm$ (s.e.) $7{\cdot}5$ mAmp/ml. This rose to a peak of $77{\cdot}3 \pm 11{\cdot}4$ mAmp/ml within 2 hours of the injection.

One to three months after yttrium implant pituitary ablation for advanced breast cancer, patients received a similar stimulation of residual prolactin cell function. This was in addition to the standard test of growth hormone response to insulin reduced hypoglycaemia (Stewart *et al.*, *J. Endocr.*, 1971, **50**, 41). The two tests have been compared and both have demonstrated positive responses in the case of incomplete pituitary ablation, as proven by post-mortem histology of the gland.

The chlorpromazine test is simple and possibly safer than the standard test in hypophysectomized patients.